# An interdigit signalling centre instructs coordinate phalanx-joint formation governed by 5′Hoxd–Gli3 antagonism

**DOI:** 10.1038/ncomms12903

**Published:** 2016-10-07

**Authors:** Bau-Lin Huang, Anna Trofka, Aki Furusawa, Jacqueline L. Norrie, Adam H. Rabinowitz, Steven A. Vokes, M. Mark Taketo, Jozsef Zakany, Susan Mackem

**Affiliations:** 1 Cancer and Developmental Biology Laboratory, CCR, NCI, Frederick, Maryland 21702, USA; 2 Department of Molecular Biosciences and Institute for Cellular and Molecular Biology, University of Texas at Austin, Austin, Texas 78712, USA; 3 Department of Pharmacology, Graduate School of Medicine, Kyoto University, Yoshida-Konoe-cho, Sakyo-ku, Kyoto 606–8501, Japan; 4 Department of Genetics and Evolution, University of Geneva, Geneva 4 1211, Switzerland

## Abstract

The number of phalanges and joints are key features of digit ‘identity' and are central to limb functionality and evolutionary adaptation. Prior chick work indicated that digit phalanges and their associated joints arise in a different manner than the more sparsely jointed long bones, and their identity is regulated by differential signalling from adjacent interdigits. Currently, there is no genetic evidence for this model, and the molecular mechanisms governing digit joint specification remain poorly understood. Using genetic approaches in mouse, here we show that functional *5′Hoxd–Gli3* antagonism acts indirectly, through Bmp signalling from the interdigital mesenchyme, to regulate specification of joint progenitors, which arise in conjunction with phalangeal precursors at the digit tip. Phalanx number, although co-regulated, can be uncoupled from joint specification. We propose that *5′Hoxd* genes and *Gli3* are part of an interdigital signalling centre that sets net Bmp signalling levels from different interdigits to coordinately regulate phalanx and joint formation.

## Abstract (web summary)

The molecular mechanisms governing digit joint specification are poorly understood. Here, the authors identify the 5′Hoxd–Gli3 balance as a key regulator of the net interdigital Bmp signalling level in mouse, which in turn regulates phalanx and joint formation.

## Graphical abstract

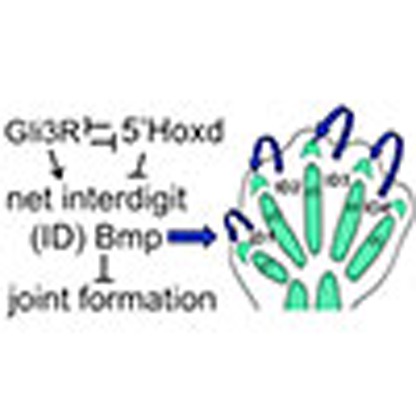

The number of digit phalanges and joints varies over a wide range in vertebrates during evolutionary adaptation for different functions, ranging from extreme hyperphalangy in marine mammal flippers to highly reduced formulas in some bat wing digits^1^,^2^,^3^. In the early limb bud, Sonic hedgehog (Shh) plays a central role in specifying digit identity, for which phalanx/joint number serves as a major hallmark^4^; however, the steps linking early anterior–posterior (A–P) patterning with later morphogenesis remain unclear. Direct evidence for ongoing regulation comes from pioneering work in the chick, demonstrating that late interdigital mesenchyme signals instruct digit ‘identity' at the stage when digit condensations first appear^5^,^6^. Exposure to more posterior interdigits can reprogramme the number of phalangeal elements that form to more posterior identity (increased number), and, conversely, exposure to more anterior interdigits produces anterior transformations (decreased number). Graded interdigital Bmp signalling has been proposed to generate this effect^5^; however, this remains controversial and genetic studies in mouse have so far failed to support such a role^7^. Furthermore, how A–P patterning inputs become translated into proximo-distal digit differences during periodic phalanx-joint formation remains enigmatic.

Joint progenitors (interzones), along with cartilage elements, arise from Sox9+ progenitors^8^, and their specification towards joint fate entails a loss of *Sox9* and initiation of *Gdf5* expression^9^,^10^,^11^,^12^. Analysis of mouse mutants, as well as manipulation of signalling in chick, have identified both Wnt and Bmp pathways as playing pivotal roles in directing this process. Canonical Wnt signalling plays an essential role in interzone formation and can induce ectopic interzones^13^,^14^. Conversely, Bmp pathway activation suppresses joint formation^15^,^16^,^17^, whereas mutants with reduced Bmp activity, such as *Bmpr1b* (ref. 18) and *Gdf5* (ref. 10) have reduced phalanges and expanded digit joint progenitors. c-Jun directs expression of canonical *Wnts* in early interzones^19^; however, this follows *Gdf5* expression onset, suggesting that earlier events upstream of Wnt activity direct interzone specification.

*5′Hox* genes (*Hoxd11-d13* and H*oxa13*) play key roles in A–P patterning^20^,^21^,^22^,^23^, and their expression spans early through late stages of limb development; however, how they act is still poorly understood. Defects in digit joint formation have been demonstrated in *5′Hoxd*^24^ as well as in *Hoxa13* (ref. 23) mutants. Previous work focused mainly on descriptive characterization and later bone phenotypes, and consequently the timing and mechanisms by which *5′Hox* genes act to regulate these late emerging features of digit identity remain obscure. Gli3, as a major Shh pathway transducer, also plays a prime role in digit patterning and *5′Hoxd–Gli3* genetic interactions alter both digit pattern and number^25^,^26^,^27^. Conditional *Gli3* deletion has revealed important roles at both early and later stages of digit development, particularly in promoting mesenchymal condensation to form digits^28^,^29^. However, the basis for altered proximal phalanges with broad gaps, suggesting expanded joints in the *Gli3* mutant, has not been determined.

In this study, we analyse the *5′Hoxd*^*−/−*^ (*Hoxd*^Del(11–13)/Del(11–13)^; *Hoxd11-d13* deleted)^20^ and *Gli3*^*−*/*−*^ (*Gli3*^XtJ/XtJ^)^30^,^31^ mutants and show that the *5′Hoxd–Gli3* balance regulates digit interzone specification in conjunction with phalanx formation, acting through Bmp pathway modulation. This regulation occurs non-autonomously from the interdigits, providing a potential mechanism for generating different Bmp input levels along the distal A–P limb bud via graded *5′Hoxd* distribution. We also show that the interzones form in close association with phalangeal primordia (phalanx-forming region) at the digit tips, and have reduced Bmp responsiveness that is governed by *5′Hoxd–Gli3* balance. These results provide the first genetic evidence for digit identity regulation by a late interdigit signalling centre and highlight a new role for *5′Hoxd* genes and *Gli3* as a component of this signalling centre.

## Results

### Opposing joint phenotypes in *5′* *Hoxd* ^*−*/*−*^ and *Gli3* ^*−*/*−*^ digits

**Figure 1:**
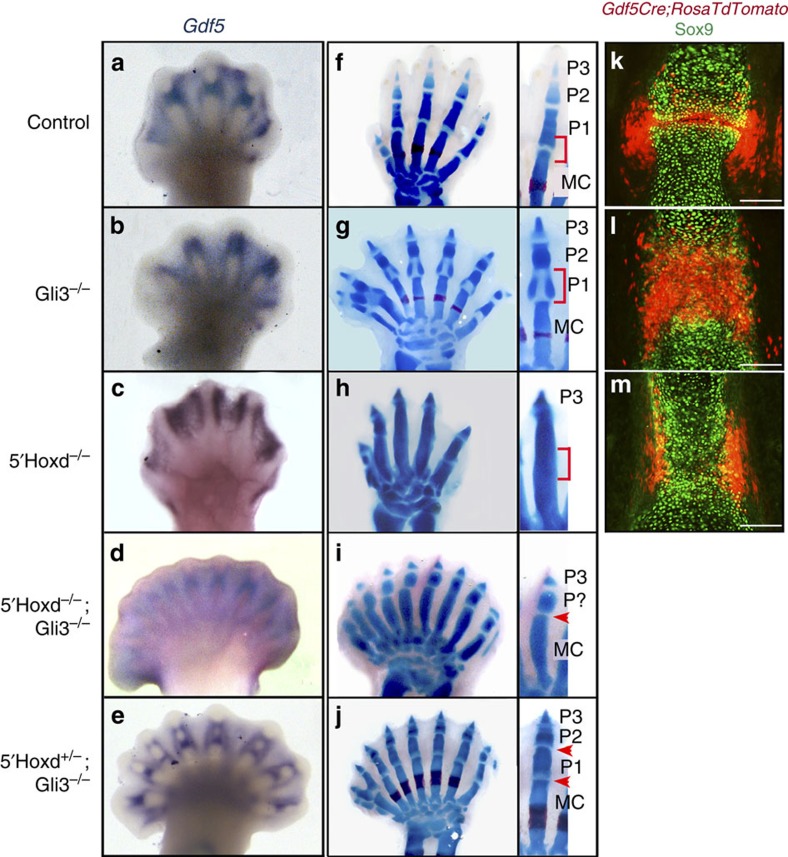
Dose-dependent *Gli3-5′Hoxd* interaction regulates digit joint formation. (**a**–**e**) *Gdf5* RNA expression (E13–13.5) in early interzones in control (*Gli3*^+/*−*^) and different mutant genotypes (indicated to left); interzones are expanded in *Gli3*^*−*/*−*^, absent in *5′Hoxd*^*−*/*−*^ digit condensations and more normal width in *Gli3*^*−*/*−*^ upon *5′Hoxd* gene dosage reduction. (**d**,**e**) are slightly later stage than panels (**a**–**c**). (**f**–**j**) E17.5 skeletal stains show digit morphologies in corresponding control and different mutant genotypes with comparable changes in digit joints. Brackets in insets to left show digit joint regions examined in **k**–**m**, and sites of joint restoration (arrowheads) in compound mutants in **i**,**j** (*n*=10/10 for *5′Hoxd*^*−*/*−*^;*Gli3*^*−*/*−*^; *n*=16/16 for *5′Hoxd*^+/*−*^;*Gli3*^*−*/*−*^). Note that *5′Hoxd*^*−*/*−*^;*Gli3*^*−*/*−*^ digits are all biphalangeal; the internal phalanx is designated ‘P?' to indicate uncertain identity (P1 versus P2). (**k**–**m**) Sox9 immunofluorescence (green) on sections of E17.5 digit cartilage elements from control, *Gli3*^*−*/*−*^ and *5′Hoxd*^*−*/*−*^ embryos also expressing *Gdf5Cre* and *RosaTdTomato* reporter alleles^35^ to mark interzone descendant cells (red). Scale bar, 100 μm for **k**–**m**. P1, 2, 3, phalanx 1, 2, 3; MC, metacarpal.

At skeletal stages (E14.5 and later), *5′Hoxd*^*−/−*^ (*Hoxd11-d13* deleted)^20^ mutant embryos have biphalangeal digits with poorly formed or absent joints in the forelimb, as previously reported^24^,^26^,^32^. In particular, metacarpal–phalangeal joints are generally absent in digits 3 and 4, and occasionally absent in digits 2 and 5 (15–30% frequency, respectively, Fig. 1f–j, Supplementary Fig. 1), although a non-cavitated pseudoarthrosis forms postnatally^32^ (see Supplementary Fig. 1). In contrast, *Gli3*^*−*/*−*^ (refs 30, 31) mutants appear to have a vertically expanded joint region within the proximal (P1) phalanges of digits (Fig. 1f–h). This expanded zone extends variably into, and sometimes replaces most of the P1 element (see Supplementary Fig. 1 for E16.5–18.5), particularly in the centre of the phalanx, leaving a peripheral rim of cartilage. Because a residual P1 element is commonly present, we interpret the *Gli3*^*−/−*^ mutant as having triphalangeal digits with conversion or replacement of part of P1 by joint interzone cells, rather than having truly biphalangeal digits with a complete loss of P1 (refs 26, 32).

The impression of opposing digit joint loss and expansion phenotypes in *5′Hoxd*^*−/−*^ and *Gli3*^*−/−*^ mutants, respectively, was supported by corresponding changes in nuclear β-catenin and Sox9 levels in mutant presumptive joint regions at E17.5 (Supplementary Fig. 2a). We focused our analysis on forelimb because the *5′Hoxd*^*−*/*−*^ hindlimb phenotype is more variable; the *Gli3*^*−*/*−*^ phenotype is similar in both limbs. Expression of the earliest joint progenitor (interzone) marker, *Gdf5* (refs 19, 33), indicated that these phenotypic changes begin early, when interzones first appear. At E12.5 when the first proximal phalangeal interzone has formed and is marked by a band of *Gdf5* expression in control digits, expression was absent from *5′Hoxd*^*−*/*−*^ digital rays and conversely expanded in *Gli3*^*−*/*−*^ digital rays (Fig. 1a–c). Genetic lineage tracing of joint progenitors using *Gdf5Cre* (refs. 9, 34) to activate the *RosaTdTomato* reporter^35^ showed an altered interzone specification in these mutants (Fig. 1k–m). *Gdf5*+ descendants were markedly expanded in the *Gli3*^*−*/*−*^ digit P1 regions, and were absent from *5′Hoxd*^*−*/*−*^ digit cartilages, although perichondrial *Gdf5*+ descendants persisted surrounding presumptive joint regions, suggesting that regulation of some positional cues may be partly preserved. These results indicate that interzone progenitors are expanded in *Gli3*^*−*/*−*^ P1 joint regions, but are not specified properly in *5′Hoxd*^*−*/*−*^ digits. Cell survival and proliferation were unaltered (Supplementary Fig. 2b), suggesting that a primary change in cell fate is more likely responsible, although proliferation rates were in accord with altered chondrogenic versus interzone fate (lower rates in the latter). The strikingly opposed interzone phenotypes raised the question of whether antagonistic *5′Hoxd–Gli3* interaction directs digit joint formation.

### Net *Gli3–5′Hoxd* dosage controls digit joint formation

To test whether *5′Hoxd* genes and *Gli3* interact genetically in this process, digit joint formation was examined in compound mutant embryos. Normal joint formation was restored in *5′Hoxd*^*−*/*−*^; *Gli3*^*−*/*−*^ digits, although digits were bi-phalangeal (Fig. 1i, *n*=10/10), as seen previously^32^. This result indicates that the 5′Hoxd–Gli3 balance affects phalanx number as well as interzone formation, and, although usually coupled, interzone formation and phalanx number are determined independently (compare Fig. 1h–j; in compound mutants normal joint formation can be rescued either with or without restoration of normal phalanx number depending on relative *Gli3* and *5′Hoxd* dosage). Strikingly, deletion of a single genomic copy of the *5′Hoxd* locus corrected the *Gli3*^*−*/*−*^ P1 phenotype and preserved normal triphalangeal digit morphologies (Fig. 1j, *n*=16/16). In early interzones, *Gdf5* RNA bands in *5′Hoxd*^*−*/*−*^;*Gli3*^*−*/*−*^ and in *5′Hoxd*^+/*−*^;*Gli3*^*−*/*−*^ digits were also more restricted and discrete compared with *Gli3*^*−/−*^ (Fig. 1b,d,e). At the stage of digit interzone formation onset (∼E12.5), RNA and protein levels of Gli3 were unchanged in the *5′Hoxd*^*−*/*−*^ handplate, and, similarly, Hoxd13 levels were unaffected at this stage in *Gli3*^*−*/*−*^ interdigits (Supplementary Fig. 3). This result is consistent with the normal coexpression of *5′Hoxd* and *Gli3* genes in interdigits after the late distal expansion of *5′Hoxd* expression^25^, and indicates that 5′Hoxd and Gli3 proteins do not act hierarchically at this stage in interdigital mesenchyme, but rather the balance between 5′Hoxd–Gli3 levels controls digit joint formation.

### *5′Hoxd* and *Gli3* genes act non-autonomously from interdigits

**Figure 2:**
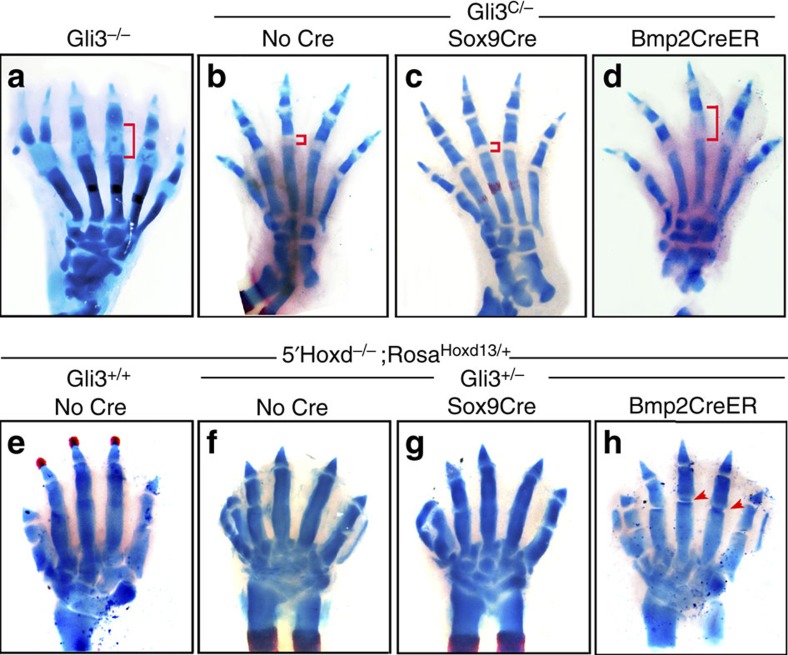
*5′Hoxd* and *Gli3* functions are required in interdigital mesenchyme for normal joint formation. (**a**–**d**) E17.5 skeletal stains show expanded P1 joint (brackets) phenotype in germline *Gli3*^*−*/*−*^ (**a**) compared with control *Gli3*^C/*−*^ digits (**b**, *n*=16/16). Selective deletion of the *Gli3 floxed* allele (*Gli3*^C/*−*^)^29^ in interdigits with *Bmp2CreER* (3 mg tamoxifen at E11.5) produces expanded P1 joints similar to *Gli3*^*−*/*−*^ (**d**, *n*=11/17), but deletion by *Sox9Cre* in interzone/chondroprogenitors does not (**c**, *n*=0/11). (**e**–**h**) E17.5 skeletal stains show lack of digit joints in *5′Hoxd*^*−*/*−*^ control (**e**), and similarly in *5′Hoxd*^*−*/*−*^;*Gli3*^+/*−*^ (**f**, *n*=4/4). Selective activation of a conditional (*floxed*) *RosaHoxd13* transgene in interdigits by *Bmp2CreER* (3 mg tamoxifen at E11.5) restores joint formation (arrowheads) in *5′Hoxd*^*−*/*−*^;*Gli3*^+/*−*^ digits (**h**, *n*=5/7), but *RosaHoxd13* activation in interzone/chondroprogenitors by *Sox9Cre* does not (**g**, *n*=0/16).

To determine the tissue requirements for *Gli3* and *5′Hoxd* function during interzone formation, joint phenotypes were examined in conditional alleles using selective Cre drivers. Selective deletion of *Gli3* from interzone- and chondoprogenitors using *Sox9Cre* (ref. 8) failed to produce an abnormal joint phenotype (*n*=0/11). In contrast, selective *Gli3* deletion from interdigital mesenchyme using *Bmp2CreER* (ref. 36) at E11.5 resulted in expanded P1 joints mimicking the germline mutant phenotype (Fig. 2a–d *n*=11/17; Supplementary Fig. 4a, *n*=8/14). The P1 phenotype was far more penetrant in hindlimb than forelimb, possibly owing to differences in Cre expression levels and timing of tamoxifen treatment (E11.25 or later used to limit recombination to interdigits)^36^.

Perdurance of Hoxd13 and Hoxd12 complicated the use of a conditional *5′Hoxd* allele to evaluate selective loss of function owing to a significantly reduced efficiency of recombination of the ∼30 kbp *flox*ed region in the *5′Hoxd* genomic locus, and possibly also protein half-life. An alternative approach was used to selectively restore Hoxd13 function in the *5′Hoxd*^*−*/*−*^ mutant. Selective activation of a conditional *RosaHoxd13* transgene in interzone- and chondroprogenitors using *Sox9Cre* failed to restore interzone formation in *5′Hoxd*^*−*/*−*^ digits (Fig. 2g, *n*=0/16). However, selective activation in interdigits using *Bmp2CreER* partially restored joint formation in *5′Hoxd*^*−*/*−*^ (Supplementary Fig. 4d, *n*=2/2); notably, the digits remained biphalangeal, possibly owing to low transgene expression (∼25% of normal endogenous Hoxd13 level, Supplementary Fig. 4c). Reduced *Gli3* dosage (*Gli3*^+/*−*^) further improved the efficacy of this rescue (Fig. 2e–h, *n*=5/7), supporting a role for proper 5′Hoxd–Gli3 balance during digit joint formation, but had no impact when the *RosaHoxd13* transgene was activated by *Sox9Cre*. These results indicate that both *5′Hoxd* and *Gli3* functions are required non-autonomously in the interdigital mesenchyme to regulate joint formation and suggest that they may act as part of an interdigit signalling centre that instructs final digit ‘identities'.

### *5′Hoxd–Gli3* dosage sets net interdigit Bmp signalling level

**Figure 3:**
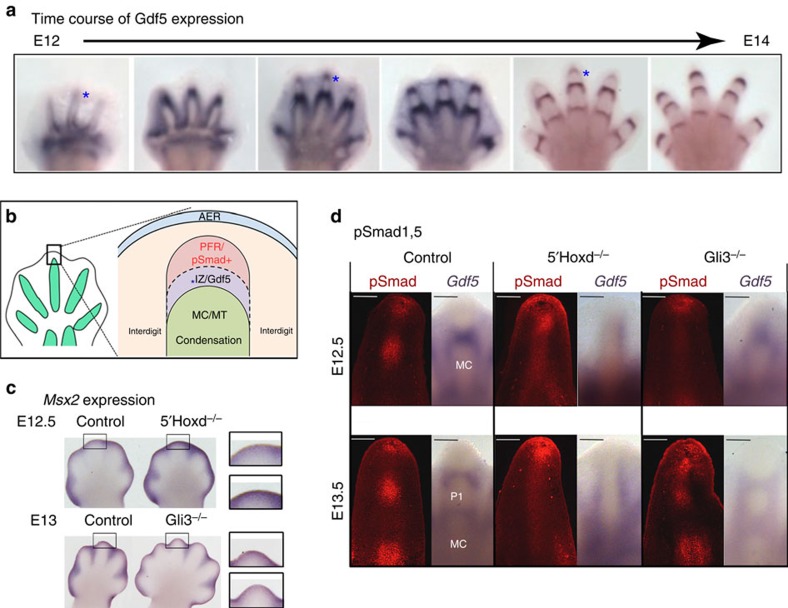
*Gdf5* expression–Bmp activity relationship in wild type compared to *5′Hoxd* and *Gli3* mutant digits. (**a**) *Gdf5* expression during digit formation (E12–E14). New bands of *Gdf5* (*) appear sequentially at the distal digit tips in proximity to the PFR (schematic in **b**). (**b**) Schematic of PFR (ref. 5), showing proposed relationship of newly forming *Gdf5*+ interzone with PFR (nascent phalanges recruited from sub-AER progenitor pool) and the proximal digit condensation. (**c**) Expression of direct Bmp target *Msx2* around distal digit tips in control (wild type) and *5′Hoxd*^*−*/*−*^ and *Gli3*^*−*/*−*^ digits at E12.5 or E13. Boxed regions shown towards right highlight differences in *Msx2* in digit tip between control (upper insets) and mutant (lower insets) for *5′Hoxd*^*−*/*−*^ and *Gli3*^*−*/*−*^ digits. (**d**) *Gdf5* RNA expression compared with pSmad1,5 (pSmad) immunofluorescence in the same digit from contralateral limb buds of control, *5′Hoxd*^*−*/*−*^ and *Gli3*^*−*/*−*^ embryos at E12.5 and E13.5. Zones of reduced pSmad activity correspond to positions of *Gdf5+* interzones and are expanded in *Gli3*^*−*/*−*^ but reduced in *5′Hoxd*^*−*/*−*^ digits. Scale bar, 100 μm for each image. IZ, interzone; MC, metacarpal; MT, metatarsal; P1 phalanx 1.

The timing and selective interdigit requirement for *5′Hoxd* and *Gli3* function suggested an early role coincident with the first appearance of phalangeal precursors, and before interdigital apoptosis and regression. Lineage-tracing experiments in chick have shown that phalanges arise as discrete elements from distal sub-apical ectodermal ridge (AER) progenitors in a ‘phalanx-forming region' (PFR), rather than via segmentation from a single precursor condensation^5^. This mechanism of phalanx formation implies that interzone formation is closely coupled to the origin of discrete phalanges. Sox9 expression and pSmad1,5 activation at the digit tips in a zone consistent with a PFR have also been previously demonstrated in mouse^37^; however, the relationship of interzone formation to the PFR has not been directly assessed in either mouse or chick. We evaluated the time course of *Gdf5* expression during phalanx formation (E12–E14; Fig. 3a) and, indeed, bands of expression first appear very near the distal digit tips and become proximally displaced over time during elongation. This progression suggests that interzones are specified coordinately in the PFR region in conjunction with nascent phalangeal elements (see Fig. 3b) in response to interdigit signals.

The canonical Wnt and Bmp pathways are both highly active during stages of digit appearance, with multiple ligands and secreted antagonists for each pathway expressed in interdigital mesenchyme^38^,^39^. Canonical Wnt signalling plays a central and well-documented role in early stages of joint formation^13^,^14^, and we first checked whether increasing β-catenin activity in Sox9+ interzone progenitors could restore normal interzone formation in *5′Hoxd*^*−*/*−*^ digits. An inducible *Sox9CreER*^40^ was used to activate β-catenin to lessen the severe inhibition of chondrogenesis known to result from very early activated Wnt signalling in nascent condensations^41^. β-catenin activation by deleting *exon 3* to generate a stabilized protein^42^ perturbed chondrogenesis in the digit cores while preserving joints in control embryos; in *5′Hoxd*^*−*/*−*^ digits, chondrogenesis was similarly altered, but with no restoration of joint formation (Supplementary Fig. 5, *n*=4/4). *Gdf5* interzone expression is not fully abolished by loss of either Wnt ligands^19^ or β-catenin^43^ in digit progenitors, but is completely absent from *5′Hoxd*^*−*/*−*^ presumptive interzone regions, suggesting that *5′Hoxd* genes may be required at an earlier step in interzone specification.

This led us to consider other signalling pathways that could play an early role in digit interzone specification. Excess Bmp activity interferes with joint formation^16^,^17^ and mouse null mutants in *Noggin*, a Bmp antagonist highly expressed in very early-stage condensations, fail to form joints and completely lack *Gdf5* expression^15^. Furthermore, Gli3 has been shown to be a key positive regulator of Bmp activity during stages when digit condensations arise^28^. We examined the Bmp activity level in *5′Hoxd*^*−*/*−*^ and *Gli3*^*−*/*−*^ around digit tips (PFR region) using several reporters for Bmp activity, including direct targets *Msx2* and *Id1*, and a Bmp-response element reporter line, *BRELacZ* (ref. 44). In wild-type controls, *Msx2* was highly expressed both in interdigits and the distal tips of E12.5 limb buds (Fig. 3c). In comparison, Bmp response was increased around *5′Hoxd*^*−*/*−*^ distal digit tips, and reduced in *Gli3*^*−*/*−*^ (Fig. 3c). Similar Bmp activity changes were inferred from *Id1* expression (both *in situ* and using quantitative PCR (qPCR; Supplementary Fig. 6a)) as well as BRElacZ activity (Supplementary Fig. 6b). However, none of these reporters are readily detected within digit condensations. To evaluate Bmp activity levels more directly in relation to forming and recently specified interzones, we examined the Bmp receptor-activated Smad effectors, phospho-Smad1,5 (pSmad). In wild-type digits, regions with reduced pSmad levels correlated directly with the positions of *Gdf5*+ interzones; in addition, a focus of high pSmad activity was present distally, near the AER (Fig. 3d; Supplementary Fig. 6c), correlating with the reported PFR location^5^,^37^. In *5′Hoxd*^*−*/*−*^ digits, no clear reduction in pSmad was seen, consistent with a lack of *Gdf5*+ zones, and, interestingly, ectodermal pSmad over digit tips was highly elevated, particularly at E12.5. Conversely, in *Gli3*^*−*/*−*^ digits, expanded zones with reduced pSmad levels correlated with broader *Gdf5*+ interzones. These results suggest that the relative 5′Hoxd–Gli3 levels modulate the net Bmp signalling level from interdigits to regulate interzone formation.

**Figure 4:**
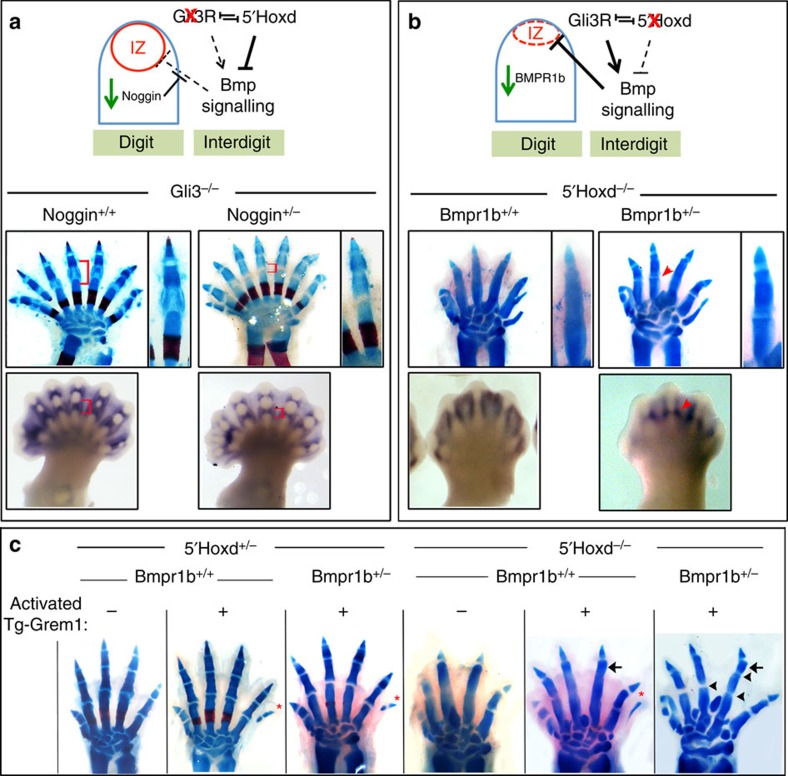
*5′Hoxd–Gli3* balance regulates coordinate joint and phalanx formation by modulating the net Bmp level. (**a**) Effect of Bmp upmodulation in *Gli3*^*−*/*−*^ digits by reduced *Noggin* dosage. Diagram shows proposed role of 5′Hoxd–Gli3 interaction in regulating net interdigit Bmp signalling and effect of reduced Gli3 input. Middle panels and enlarged insets to right show skeletal stain at E17.5 and restoration of normal P1 digit joints (brackets) when *Noggin* is reduced (*Noggin*^+/*−*^; *n*=13/13). Lower panels show similarly altered *Gdf5* interzone expression (brackets) at E13.5. (**b**) Effect of Bmp downmodulation in *5′Hoxd*^*−*/*−*^ digits by reduced *Bmpr1b* dosage. Diagram shows proposed role of 5′Hoxd–Gli3 interaction and effect of reduced 5′Hoxd input. Middle panels and enlarged insets to right show skeletal stain at E17.5 and restoration of digit joints (arrowhead) when *Bmpr1b* is reduced (*Bmpr1b*^+/*−*^; *n*=17/30 for digit 3). Lower panels show similarly altered *Gdf5* interzone expression (arrowhead) at E13.5. (**c**) Further Bmp downmodulation in *5′Hoxd*^*−*/*−*^ digits by increasing Grem1 improves phalanx as well as interzone formation. E17.5 skeletal stains show that reducing Bmp signalling more extensively in *5′Hoxd*^*−*/*−*^;*Bmpr1b*^+/*−*^ embryos by ‘activated' transgenic *RosaGrem1* (Tg-Grem1) expression (*Hoxb6CreER*+; 3 mg tamoxifen at E10.75) results in improved phalanx (arrows, *n*=11/11) and joint formation (arrowheads, *n*=2/11 for digit 3 and *n*=7/11 for digit 4). By comparison, Grem1 activation alone (*5′Hoxd*^*−*/*−*^;*Bmpr1b*^+/+^) improves phalanx formation (*n*=10/10) but does not restore joints (*n*=0/10). * indicates extra post-axial digit that forms owing to activated Tg-Grem1 (*n*=15/18 in *5′Hoxd*^+/*−*^ controls).

To test whether the *5′Hoxd*^*−*/*−*^ and *Gli3*^*−*/*−*^ digit phenotypes result from changes in Bmp signalling, we used genetic approaches to directly alter Bmp activity levels in these mutants. Because of the large number of ligands and antagonists expressed in interdigits that may act redundantly, we chose to modify Bmp responsiveness either by reducing the major receptor *Bmpr1b* (ref. 18) in digit condensations or reducing the major antagonist *Noggin*^15^ present within condensing digit mesenchyme to increase the availability of ligand locally. Reduced *Noggin* gene dosage (*Noggin*^+/*−*^) in the *Gli3*^*−*/*−*^ mutant restored normal P1 morphology and joints very efficiently (∼100%, *n*=13/13), preceded by more restricted *Gdf5*+ zones at earlier stages (Fig. 4b). Conversely, simply reducing *Bmpr1b* gene dosage (*Bmpr1b*^+/*−*^) in the *5′Hoxd*^*−*/*−*^ mutant restored the formation of digit joints with ∼56% efficiency, most commonly evident in digit 3 (Fig. 4a; *n*=17/30), and was preceded by the restoration of *Gdf5*-expressing interzones at early stages.

### Bmp levels regulate coordinate phalanx–interzone formation

**Figure 5:**
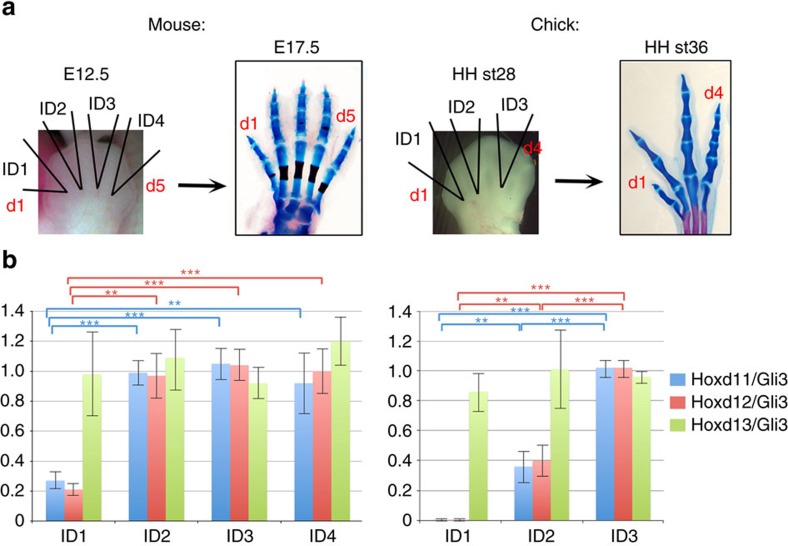
Total 5′Hoxd:Gli3 stoichiometry in different A–P interdigits correlates with phalanx number. (**a**) Diagram of individual interdigits dissected for qPCR in E12.5 mouse hindlimb and HH stage 28 chick leg, and normal digit phenotypes of E17.5 mouse hindlimb (A–P phalanx numbers 2-3-3-3-3 from d1 to d5) and HH stage 36 chick leg (A–P phalanx numbers 2-3-4-5 from d1 to d4). (**b**) Graphs of normalized Hoxd/Gli3 expression ratios in different mouse and chick interdigits (ID; from qPCR analyses of three independent sample sets; see Methods for details). Error bars represent s.d. Changes in both Hoxd11/Gli3 and Hoxd12/Gli3 are highly significant between ID1 and other interdigits (ID2–ID4) in mouse, and between all interdigit pairs (ID1–ID2–ID3) examined in chick, using Student's two-tailed *t-*test (**P<0.01, ***P<0.001). In mouse, ID2–ID4 showed uniform expression ratios, consistent with an invariant triphalangeal morphology. The posterior-most digit (d5 in mouse or d4 in chick) was not included in analysis because evidence in chick indicates that phalanx number regulation in the posterior-most digit is complex and is not solely dependent on interdigit signals^5^.

**Figure 6:**
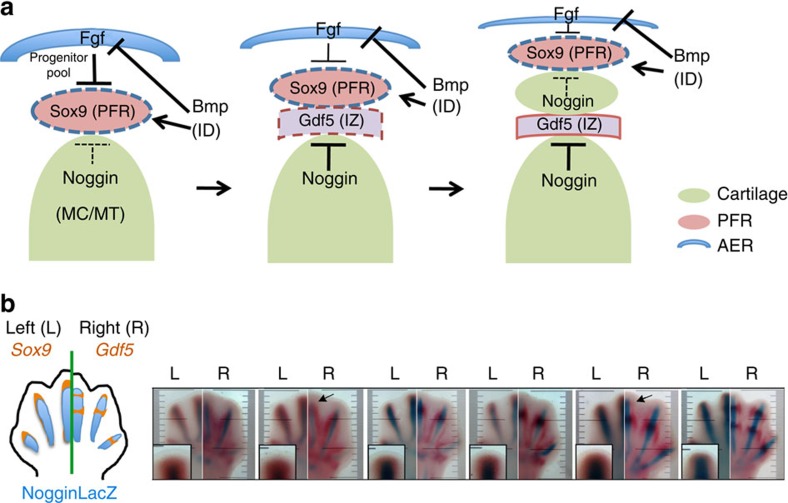
Model for periodic interzone (IZ) specification based on local Gdf5–Noggin induction timing. (**a**) Model for periodic digit IZ specification incorporating inputs from distal AER/Fgf, net interdigit (ID) Bmp levels and Noggin signals from proximal condensations. AER/Fgf maintains an uncommitted progenitor pool, inhibiting formation of the Sox9+ PFR. As cells become removed from AER influence, they become Bmp-responsive and form new pSmad+, Sox9+ phalanx progenitors (PFR) that contribute to incipient condensations. Noggin expression subsequently initiates in mature condensations proximal to the PFR region and locally suppresses Bmp/pSmad activity in adjacent PFR cells, enabling formation of a new IZ in conjunction with the PFR. Initiation of Gdf5 expression in the new IZ serves as a barrier to limit further IZ extension and preserve a PFR zone by binding to and sequestering Noggin. Bmps from IDs may also inhibit AER function, promoting regression and decline in the progenitor pool. ID, interdigit; IZ, interzone; MC/MT, metacarpal/metatarsal element; PFR, phalanx-forming region. (**b**) Temporospatial relation of *Sox9*, *Gdf5* and *Noggin* expression during phalanx formation is compatible with a Gdf5–Noggin-driven periodic interzone model. Time course (E12.5–13.5) comparing *Sox9* RNA/NogginLacZ activity (L) with *Gdf5* RNA/NogginLacZ activity (R) in contralateral limb bud pairs (L, left; R, right), as shown schematically to left. Insets show enlarged tip regions with *Sox9* expression extending distally beyond the Noggin+ zone (this distal crescent identifies a Sox9+ PFR zone). *Gdf5*+ zones first appear at digit tips (arrows) at the distal edge of the Noggin+ domains in proximal elements.

Limiting Bmp signalling by reducing *Bmpr1b* dosage in *5′Hoxd*^*−*/*−*^ digits had little effect on phalanx number. To further reduce net Bmp levels, we activated transgenic expression of the *Gremlin1* Bmp antagonist in *5′Hoxd*^*−*/*−*^; *Bmpr1b*^+/*−*^ embryos. The *Rosa*^*Grem1/+*^ transgene has been shown to induce polydactyly by modulating Bmp activity in early embryos^45^, using *Hoxb6CreER*-mediated activation. Because of the low level of gene expression from the *Rosa* promoter (see for example, Supplementary Fig. 4c), we chose to activate at E10.75 using Hoxb6CreER to ensure robust transgenic Grem1 expression by ∼E12 (even in forelimb Hoxb6CreER is active across most of the distal limb mesenchyme by E11)^46^. Activation of *Rosa*^*Grem1/+*^ by Hoxb6CreER at E10.75 modulated forelimb bud Bmp signalling, resulting in downregulated Bmp target and enhanced *Fgf8* expression (Supplementary Fig. 7), and also had a modest effect on digit number (see Fig. 4c, *). Although *RosaGrem1* activation alone (Tg-Grem1) had a modest effect on *5′Hoxd*^*−/−*^ digit phenotypes, in *5′Hoxd*^*−*/*−*^; *Bmpr1b*^+/*−*^ embryos, Tg-Grem1 both further improved joint formation and resulted in the formation of small additional phalanges in digits 3 and 4 (Fig. 4c, *n*=11/11). Taken together, these results implicate altered Bmp activity as the basis for *5′Hoxd*^*−*/*−*^ and *Gli3*^*−*/*−*^ digit joint phenotypes and suggest that Bmp levels set by the 5′Hoxd–Gli3 balance in the interdigital mesenchyme coordinately regulate both phalanx and interzone formation. Since total *5′Hoxd* levels are graded along the A–P limb bud interdigits at the same stage (by ∼E12)^25^,^47^,^48^, one can envision that differing 5′Hoxd–Gli3 stoichiometry in different interdigits could regulate formation of digits with different phalanx/interzone numbers across the limb A–P axis. In mouse, this stoichiometry normally produces either 2 (thumb) or 3 phalangeal elements, which is shifted to two elements by loss of *5′Hoxd* activity (Fig. 1i,j). A more extensive range in 5′Hoxd–Gli3 stoichiometry would be expected to produce a greater variation in phalanx number. To test this prediction, we quantified the steepness of the interdigit *5′Hoxd* gradient in chick hindlimb, where the number of elements and joints steadily increase along the A–P axis, along with greater variation in spacing of the elements^1^. In fact, while the ratio of *Hoxd13/Gli3* transcripts is relatively constant in different A–P chick interdigits similar to mouse, the ratio of *Hoxd11/Gli3* and of *Hoxd12/Gli3* increases dramatically from interdigit 1 to 2 to 3 in chick, but only between interdigits 1 and 2 in mouse (Fig. 5). Our results concur with the conclusion of previous work that interdigital mesenchyme functions as a late signalling centre to regulate digit identity^6^, and we propose that different interdigit Bmp levels are set by varying Hoxd/Gli3 stoichiometry. Bmps, by promoting the formation of a Sox9+ PFR (see Fig. 6), are essential for phalanx formation^5^,^37^, but in excess may simultaneously deplete the progenitor pool and lead to reduced phalanx number, as well as suppressing interzone formation.

### Periodic interzone formation via Bmp–Noggin modulation

A major question raised by these results is how periodic interzone formation might be specified by a given level of interdigit Bmp signalling that does not appear to oscillate (although pSmad response does). AER/Fgf signals in conjunction with Wnts have been shown to maintain limb mesenchymal cells in a progenitor state^49^, which can, upon withdrawal of these signals, enter the chondrogenic pathway in response to Bmps from adjacent interdigits. To establish periodicity, a proximal signal would also be required to inhibit Bmp responsiveness in PFR cells and allow them to adopt an interzone fate. In fact, at later stages, Noggin expressed at the edges of cartilage elements has been shown to play an analogous role in the formation of articular cartilage by providing local insulation from Bmp signals^50^. Given the high Noggin expression in early-stage digit condensations, we speculated that Noggin could play a similar role during coupled phalanx–interzone formation in the PFR. As outlined in Fig. 6a, when sub-AER progenitor cells become removed from ectodermal Fgf and Wnt influence during distal outgrowth, they become Bmp-responsive and begin to express Sox9, creating a PFR zone. The more mature proximal condensation begins to express Noggin, which locally suppresses Bmp response, enabling the formation of a new interzone at the proximal edge of the PFR. Gdf5 expression is then initiated within the newly formed interzone at the proximal PFR edge and serves to limit the interzone extent by acting as a sink to prevent the further distal spread of Noggin into the PFR. Such a role is supported by *Gdf5* mutant phenotypes in mouse^10^, as well as human missense mutations in *Gdf5* (ref. 51).

Indeed, the high efficiency with which reduced *Noggin* dosage (*Noggin*^+/*−*^) is able to restore normal joint formation in *Gli3*^*−*/*−*^ digits is also consistent with this model. Such a model requires a lag in *Noggin* expression relative to *Sox9* activation in the PFR, and initiation of *Gdf5* expression in close proximity with the PFR border at which *Noggin* expression begins. We checked the relative timing and spatial relationship between *Sox9*, *Noggin* and *Gdf5* expression in digit tips at different times during phalanx formation (E12.5–13.5; Fig. 6b). In fact, *Sox9* expression extended to within ∼40 μm of the AER and distally beyond Noggin+ cells at all stages examined, indicating that *Noggin* expression lags behind *Sox9* activation distally in the PFR. We also examined *Gdf5* relative to *Noggin* expression in contralateral digit tips during interzone formation; *Gdf5* was first detected at the distal border of *Noggin*+ cells (Fig. 6b). These observations are consistent with a model in which the Gdf5+ interzone is specified at the proximal PFR edge by an adjacent Noggin+ condensation that locally intercepts Bmp activity. Implicit in this model is a requirement for expansion, enabling the spatial modulation of different signalling inputs on PFR cells over time. The extent of the lag between activation of *Sox9* and subsequent *Noggin* expression in condensations arising in the PFR could determine the relative interzone–phalanx spacing.

## Discussion

The model proposed above (Figs 4a,b and 6a) supports previous work in chick showing that interdigit signalling could affect phalanx formation and regulate final ‘digit identity' at late stages^6^, and identifies novel roles for 5′Hoxd and Gli3 in control of interdigit signalling, thereby linking early with late patterning events. We have found that net Bmp signalling level plays a key role in regulating phalanx–interzone formation and propose that different set points for net Bmp activity could provide a basis for formation of different A–P digit types governed by differing Hoxd–Gli3 stoichiometry. Gli3 has been shown to upregulate net Bmp levels in the limb^50^, in part via *Grem1* repression^28^,^52^, particularly in the anterior limb bud where the P1 joint expansion phenotype is often severest (Fig. 1g and Supplementary Figs 1c and 4a). Direct binding of 5′Hox members (Hoxa and Hoxd) to the regulatory domains of several Bmps has been demonstrated and implicated in positively regulating their expression^53^,^54^,^55^. Notably, these studies were largely carried out in osteochondrogenic cells and may be more relevant to late stages and perichondrial differentiation (where *Bmp* expression appears to be 5′Hox-dependent)^24^, highlighting the complexity and stage/cell-type dependence of Bmp regulation by 5′Hoxd proteins. Regulation of Bmp antagonists, particularly Grem1, by Hox members has also been demonstrated, but attributed largely to more 3′ members of the cluster such as Hox9 paralogues^26^,^56^. In the context of interdigit signalling, it is possible that 5′Hoxd proteins affect the net Bmp signalling level very indirectly, and may even act largely by binding to and sequestering or inhibiting Gli3-repressor function^25^.

Bmps have previously been both proposed and disputed to play a central role in digit phalanx formation based on gain-of-function studies in chick and genetic analysis in mouse. Bmp levels were proposed to play a positive role in regulating phalanx formation from the PFR in chick^5^. In these studies ectopic Noggin protein reduced phalanx number; however, direct Bmp application resulted in severe digit truncations (presumably related to a side effect of AER inhibition). The effect of Noggin could also be explained by complete inhibition of distal chondrogenesis^14^,^57^, rather than a specific effect on phalanx formation. Similarly, phalangeal loss seen in mutants with extreme reduction in Bmp activity may reflect either a general inhibition of chondrogenesis (for example *Bmpr1b*^*−*/*−*^)^18^, or highlight a role in joint formation (marked expansion in interzone fate). Our results together with these previous studies suggest that varying observed Bmp effects may reflect the dosage and relative Bmp balance. At the extremes, too high a Bmp level could both impede interzone formation and deplete the uncommitted progenitor pool; whereas too low a level could result in a failure of phalanx formation in the PFR, as suggested by certain mouse *brachydactyly* mutants with reduced pSmad levels^37^. In contrast to our results, prior efforts using genetic approaches to remove *Bmp* ligands have failed to provide evidence for a Bmp role in regulating digit identity^7^,^58^. However, in one study^7^, removal of the major Bmp ligands expressed in limb was limited to pairs of ligands (with analysis restricted to *Bmp2,4,7* genes). In a recent paper^58^, an interdigit-specific Cre was used to inactivate *Bmp2,4,7* conditional alleles simultaneously, but the onset of high-level Cre expression was late (E13.5) relative to the timing of phalanx–interzone regulation (E12–13, see Fig. 3a). The large number of additional Bmp-class ligands expressed further complicates such genetic analysis^39^,^59^. Interestingly, the modulation of net total Bmp activity levels by altering antagonist expression does have an effect on phalanx–interzone formation in the *5′Hoxd* mutant (Fig. 4c), but in the wild type, an expansion beyond formation of the normal three phalanges is never observed in mouse. This may reflect the robustness of homeostatic mechanisms to maintain the wild-type complement of elements, or may also be a consequence of species differences in the total duration of progenitor pool maintenance by AER function, or in the timing of expression of other factors, such as Wnts, that promote terminal phalanx formation^37^,^60^.

Differing net Bmp levels governed by the 5′Hoxd–Gli3 balance in different interdigits provides one input that determines progenitor pool size/duration by regulating exit of progenitors into the PFR, and possibly by modulating AER- and progenitor maintenance^60^,^61^. Clearly, other regulatory inputs must also play a role^37^,^60^. In forelimb digits, information on proper interzone position appears to be at least partly preserved in *5′Hoxd*^*−*/*−*^ digits (see Fig. 1m) and rescued mutant interzones are similarly positioned to controls (Figs 1i,j and 4a,b). In addition, other models, such as a Turing mechanism^39^,^62^,^63^, that could predict periodicity in interzone specification have not been excluded. It is noteworthy that both *5′Hoxd* and *Gli3* gene functions have also been implicated genetically in controlling the number of digit rays formed in mouse^63^, which has been modelled using reaction-diffusion mechanisms involving both Bmp and Wnt pathways^39^. However, in this context, *Hoxd–Gli3* roles differ somewhat, and interact synergistically rather than antagonistically. *Sox9*+ digit rays formed by ∼E11.5, whose number varies with Hoxd–Gli3 dosage, are progenitors of the more proximal metapodial elements (Fig. 3a), and their formation is regulated at an earlier stage, prior to interdigit signalling effects on PFR/interzone digit formation. Consistent with this, Gli3 removal at later stages^29^ or selectively from interdigits (Fig. 2, Supplementary Fig. 4) still affects joint formation/phalangeal phenotypes dramatically, but does not alter digit condensation number. Future work directed towards interrogating the interplay between Bmp pathway and other inputs leading to periodic interzone–phalanx formation will be required to test different models for this process and to elucidate the contribution of AER/Fgf function.

## Methods

### Mouse strains and embryo analyses

All animal studies were carried out according to the ethical guidelines of the Institutional Animal Care and Use Committee (IACUC) at NCI-Frederick under protocol #ASP-12-405. The *Bmpr1b* (ref. 18)*, Catnb*^C*-*exon3/+^ (ref. 42), *Gli3(XtJ)*^30^, Gli3[floxed]^29^, *Hoxd*^*Del(11–13)/+*^ (*5′Hoxd*^+/*−*^; ref. 20), *NogginLacZ*^15^*, RosaGrem1* (ref. 45), *BRELacZ*^44^, *Bmp2CreER*^36^, *Gdf5Cre*^34^, *Hoxb6CreER*^46^, *Sox9Cre*^8^, *Sox9CreER*^40^ and *Rosa-tdTomato*^35^ alleles used have all been reported previously. To generate the Cre-inducible, *RosaHoxd13* knock-in mouse line, a *Hoxd13* cDNA modified to include an N-terminal-epitope [3xflag] was introduced into the pBigT shuttle vector and transfected into embryonic stem cells to target the *Rosa26* locus. Targeted embryonic stem cells were blastocyst-injected, transferred to foster mothers and chimeric offspring were outbred and screened for the recombined allele^45^. For timed matings, noon on the day of post-coital plug was considered to be E0.5. For inducible Cre drivers, a single dose of 3 mg tamoxifen (in all cases) was injected intraperitoneally at the time indicated and embryos were collected, fixed in 4% paraformaldehyde and stored in 100% methanol for further analyses.

### *In situ* hybridization and LacZ staining

Enbryos were processed as described^46^,^64^. Briefly, the fixed embryos were bleached in 5% hydrogen peroxide in methanol, followed by rehydration and a brief proteinase K treatment (5–15 min, 20 μg ml^−1^). Embryos were hybridized with digoxigenin–UTP-labelled antisense riboprobes in a standard hybridization buffer containing 50% formamide, 1% SDS and 0.75 M NaCl at 70 °C overnight. Embryos were washed in hybridization buffer at 70 °C, hybridization buffer with reduced salt (0.15 M NaCl) at 50 °C and in reduced salt (50 mM NaCl) without formamide at 70 °C, and then transferred to TBST (25 mM Tris pH 7.4, 150 mM NaCl and 0.1% Tween20) for incubation with antidigoxigenin antibody (Ab) conjugated to alkaline phosphatase (1:2,000, Roche #11093274910) at 4 °C overnight. The colorimetric reaction was developed in BM Purple (Roche #11442074001) at room temperature. For β-galactosidase staining, embryos were fixed in 2% paraformaldehyde–0.2% glutaraldehyde for 1–2 h, washed in TBST and stained in 1 mg ml^−1^ XGal, 2 mM MgCl2 in TBST at 37 °C.

### Skeletal preparation

Embryos were collected and fixed in ethanol, followed by acetone dehydration. The skeletons were stained in 0.3% Alcian Blue 8GS and 0.1% Alizarin Red S in 70% ethanol containing 5% acetic acid. Stained tissues were cleared in 2% potassium hydroxide and transferred to 50% glycerol for imaging.

### Protein immunostaining analyses

Embryos were fixed in 4% paraformaldehyde for 3 h. Primary Abs against Sox9 (1:500, Millipore #AB5535) and β-catenin (1:500, BD Bioscience #610153) in TBST were applied to 5 μm paraffin sections or 10 μm OCT frozen sections and binding visualized with Alexa Fluor 488- or 594-secondary Abs (1:500, Invitrogen). For pSmad immunofluorescence, fixed limb buds were embedded in 7% low-melting agarose and 100 μm vibrotome sections were treated with anti-phosphoSmad1,5 (1:200, Cell Signaling #9516), visualized with Alexa Fluor 594 secondary Ab and imaged using confocal microscopy. Anti-Cyclin D1 (1:50, Thermo Scientific #RM9104) and Anti-Caspase 3 (1:250, Cell Signaling #9661) were detected with horseradish peroxidase-secondary Abs (Vector Labs) on paraffin sections. For immunoblots, 1% SDS lysates of distal digital plates (digit rays and interdigits) dissected from limb buds were used, and blots were probed with affinity-purified polyclonal anti-Hoxd13 (1 μg ml^−1^ final, gift from Scott Stadler), anti-Gli3 (ref. 25; 1 μg ml^−1^ final) and anti-Vinculin (1:1,000, Sigma #V4139). Band intensities were quantitated with the Image J software or with the Odyssey Li-Cor system to quantify fluorescence signals and were normalized to Vinculin; at least three independent samples were analysed for each genotype. Significance of differences was determined using the two-tailed, Student's *t*-test.

### Transcript quantitation using qPCR

Distal digital plates (digit rays and interdigits) or individual interdigits, as indicated in text, were dissected from mouse (E11.5 or E12.5) and chick (HH stage 28 (ref. 65)) limb buds and pooled for reverse transcriptase–qPCR^64^. RNA isolation (Invitrogen #AM1931), cDNA synthesis (Invitrogen #18091050) and qPCR analysis (Quanta Bioscience, #95072-012) were carried out as recommended by the manufacturers. Relative transcript levels were normalized to Vimentin and expression fold changes of each mutant were calculated relative to wild-type controls. At least three independent limb bud samples for each genotype were analysed. Significance of differences was determined using the two-tailed Student's *t*-test. All qPCR primer sequences used are listed in Supplementary Table 1.

### Data availability

The authors declare that all data supporting the findings of this study are available within the article and its Supplementary Information Files or from the corresponding author upon reasonable request.

## Additional information

**How to cite this article:** Huang, B.-L. *et al*. An interdigit signalling centre instructs coordinate phalanx-joint formation governed by 5′Hoxd–Gli3 antagonism. *Nat. Commun.* 7:12903 doi: 10.1038/ncomms12903 (2016).

## Supplementary Material

Supplementary Information Supplementary Figures 1-7, Supplementary Table 1, Supplementary References.
