# Supplementary material for: An interdigit signalling centre instructs coordinate phalanx-joint formation governed by 5′Hoxd–Gli3 antagonism
Source: Nat Commun. 2016 Oct 7;7:12903. doi: 10.1038/ncomms12903 (PMC5059757; doi:10.1038/ncomms12903)
Supplement: Supplementary Information — Supplementary Figures 1-7, Supplementary Table 1, Supplementary References. [file ncomms12903-s1.pdf]

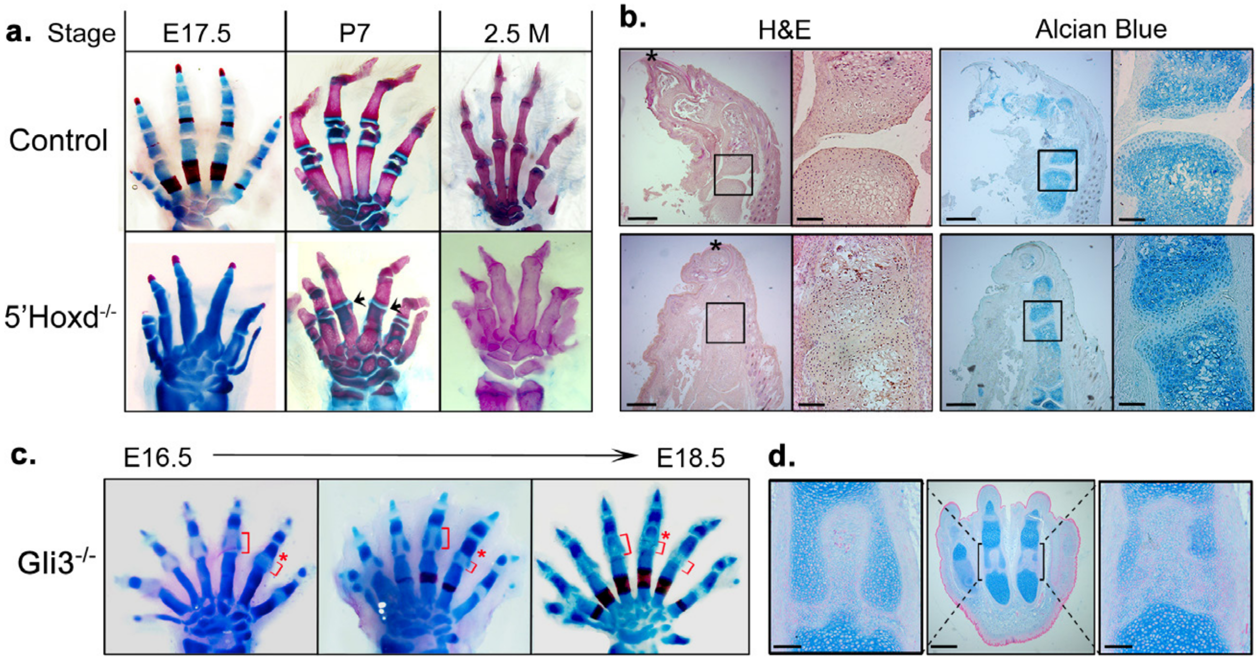

e.

5'Hoxd-/- digit phenotype classification				
Phalanges/joint phenotypes:	Digit 2	Digit 3	Digit 4	Digit 5
P3	47/47 (100%)	47/47 (100%)	47/47 (100%)	47/47 (100%)
DIP	47/47 (100%)	47/47 (100%)	47/47 (100%)	47/47 (100%)
Distinct P2, PIP-like division*	0/47 (0%)	0/47 (0%)	2/47 (4%)	0/47 (0%)
Proximal MCP joint	39/47 (83%)	0/47 (0%)	0/47 (0%)	32/47 (68%)

*see example of P2 rudiment and joint in 5'Hoxd-/- digit below

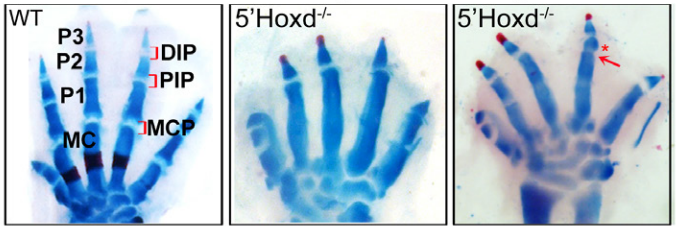

Supplementary Figure 1. Morphologic features of *5'Hoxd-/-* and *Gli3-/-* digits.

a. Stained forelimb skeletons comparing *5'Hoxd-/-* and sibling control digit features at E17.5, postnatal (P) day 7, and 2.5 months age. Arrows mark *5'Hoxd-/-* metacarpo-phalangeal (MCP) junctions lacking joints and growth plates (P7).

b. Sections of 7-week postnatal *5'Hoxd-/-* and sibling control forelimb digits stained with H&E, or alcian blue to visualize cartilage, scale bars=500 μm. Boxed MCP areas enlarged in the adjacent panels (scale bars=100 μm) show persistent cells and lack of a joint cavity in mutant. *=nail/digit tip.

c. Stained forelimb skeletons showing progression of P1 joint phenotypes in *Gli3*$^{-/-}$ embryos from E16.5 – E18.5. Brackets mark expanded MCP joints and * indicates rudiments of reduced, partial P1 phalanges. Across the limb bud, expanded MCP joints and partial P1 elements are observed ranging from reduced P1 in posterior digits to peripheral cartilage struts anteriorly.

d. Sections of alcian blue stained E17.5 *Gli3*$^{-/-}$ forelimb digits. Scale bars=500 μm. Enlarged areas (scale bars=100 μm) show expansion of MCP joint into P1 element with varying residual P1 cartilage present at digit periphery.

e. Frequency of *5'Hoxd*$^{-/-}$ forelimb digit 2-5 phenotypes (scored at E17.5; FVB/C57BL6 mixed background). Images below show wild-type control (WT) with scored features annotated, and examples of a typical phenotype (middle image), and of rare rudimentary middle phalanx (*) and PIP joint (arrow) in *5'Hoxd*$^{-/-}$ digit 4.

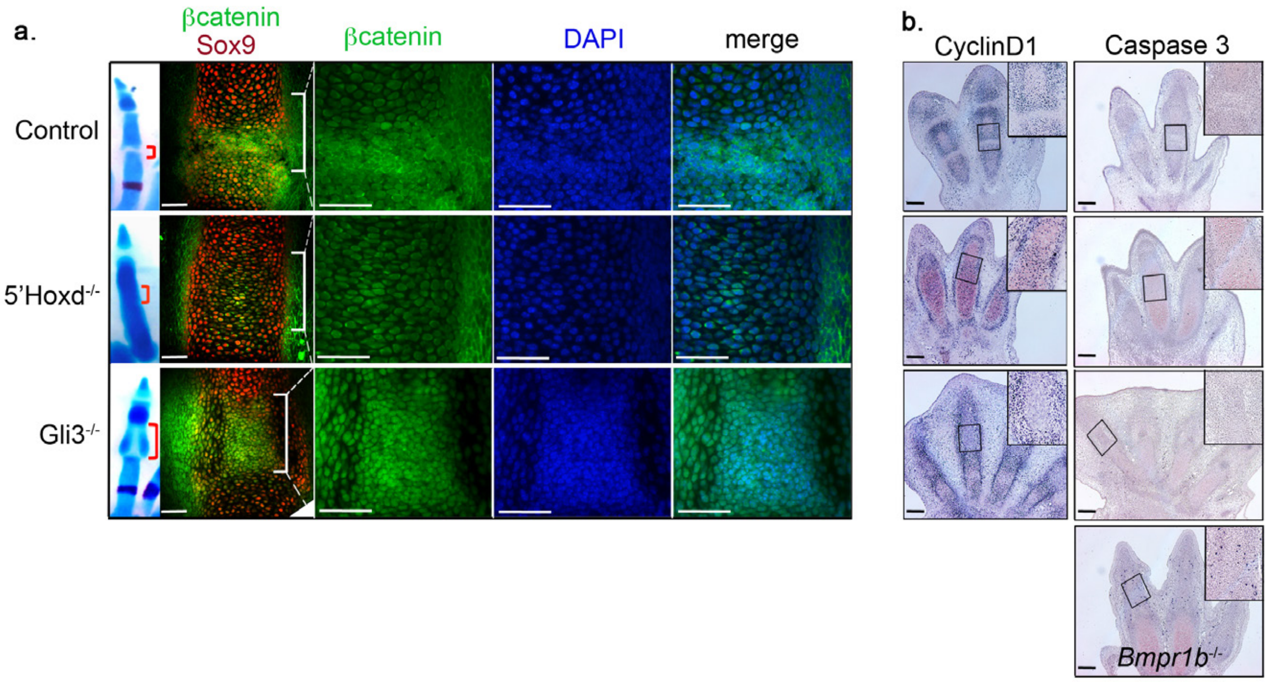

Supplementary Figure 2. ***5'Hoxd$^{-/-}$*** **and** ***Gli3$^{-/-}$*** **interzone changes are unassociated with altered proliferation or survival.**

a. Immunofluorescence for βcatenin and Sox9 shows joint changes in sections of E14.5 mutant compared to control (wild-type) digits (as indicated at left). Adjacent E17.5 skeletal panels of a single digit show approximate joint region sectioned for immunofluorescence (red bracket). White bracketed regions expanded in adjacent panels to show βcatenin localization compared to DAPI nuclear staining and overlay (merge). Scale bars=50 μm for all panels.

b. Immunohistochemistry for CyclinD1 and Caspase 3 in E13.5 sections through digits show no changes in proliferation or survival in presumptive joint regions of mutant compared to control digits; *Bmpr1b-/-* digits shown as a positive control for elevated apoptosis (Caspase 3+). Scale bars=100 μm. Boxed areas enlarged (2-fold) in insets show interzone/joint region.

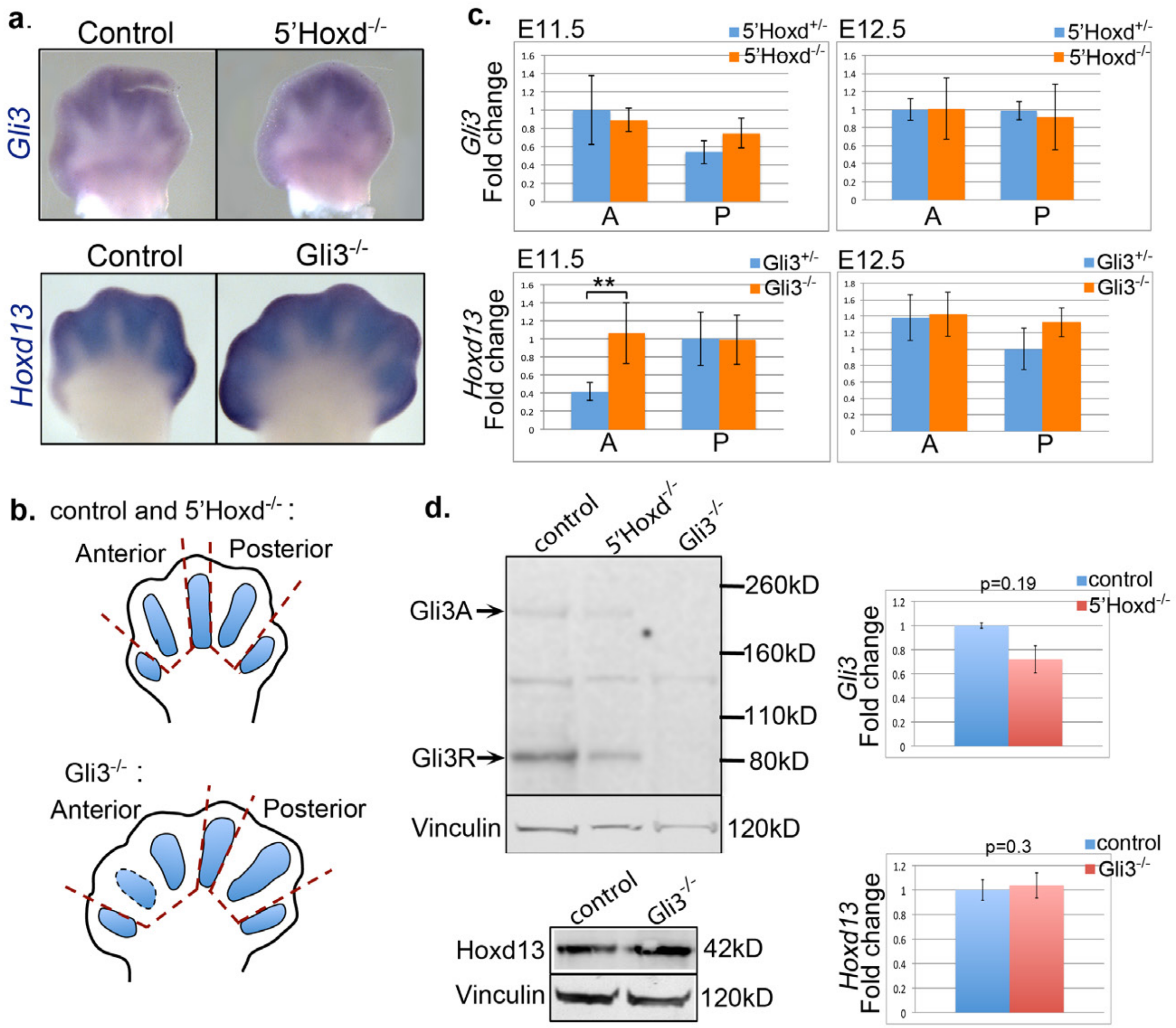

Supplementary Figure 3. ***Hoxd13*** **and** ***Gli3*** **do not repress each other's expression interdigit expression (E12.5).**

a. Whole mount RNA *in situ* analysis of *Gli3* and *Hoxd13* transcripts reveal comparable expression levels in control and mutant embryos at E12.5, as indicated on left (anterior border to left in all panels).

b. Diagrams showing anterior (A) and posterior (P) distal digital ray-interdigit regions dissected for RNA and protein analysis of mutant limb buds (in c, d), which consisted of 2-3 interdigits and 1-2 digit rays. For immunoblots (d), the posterior digital plate of *5'Hoxd*$^{-/-}$ limb buds were compared to controls, and the anterior digital plate of *Gli3*$^{-/-}$ was compared to controls, reflecting the region of highest expression and strongest respective mutant

phenotype for each factor.

c. qPCR analysis of *Gli3* or *Hoxd13* RNA levels in control and mutant embryos (at E12.5 compared to E11.5) show no significant changes in *Gli3* expression in *5'Hoxd*$^{-/-}$ digital plate (n=3) and no significant change of *Hoxd13* expression in *Gli3*$^{-/-}$ digital plate at E12.5 (n=4). In contrast, as previously documented[1], E11.5 *Hoxd13* expression is precociously expanded in the anterior limb bud (late phase 3 expression[2]) in *Gli3*$^{-/-}$ embryos (**p=0.02, n=4).

d. Immunoblot of *Gli3* or *Hoxd13* protein in control and mutant embryos at E12.5. Anti-Vinculin was used as a loading control for normalization. Graphs to right of blot bands quantitated using Image J software show no significant change of Gli3 protein level in posterior *5'Hoxd*$^{-/-}$ digital plate (n=3), and no significant change of Hoxd13 protein level in anterior *Gli3*$^{-/-}$ digital plate (n=3). p values determined using 2-tailed Student's t test, and error bars represent s.d. (in **c, d**).

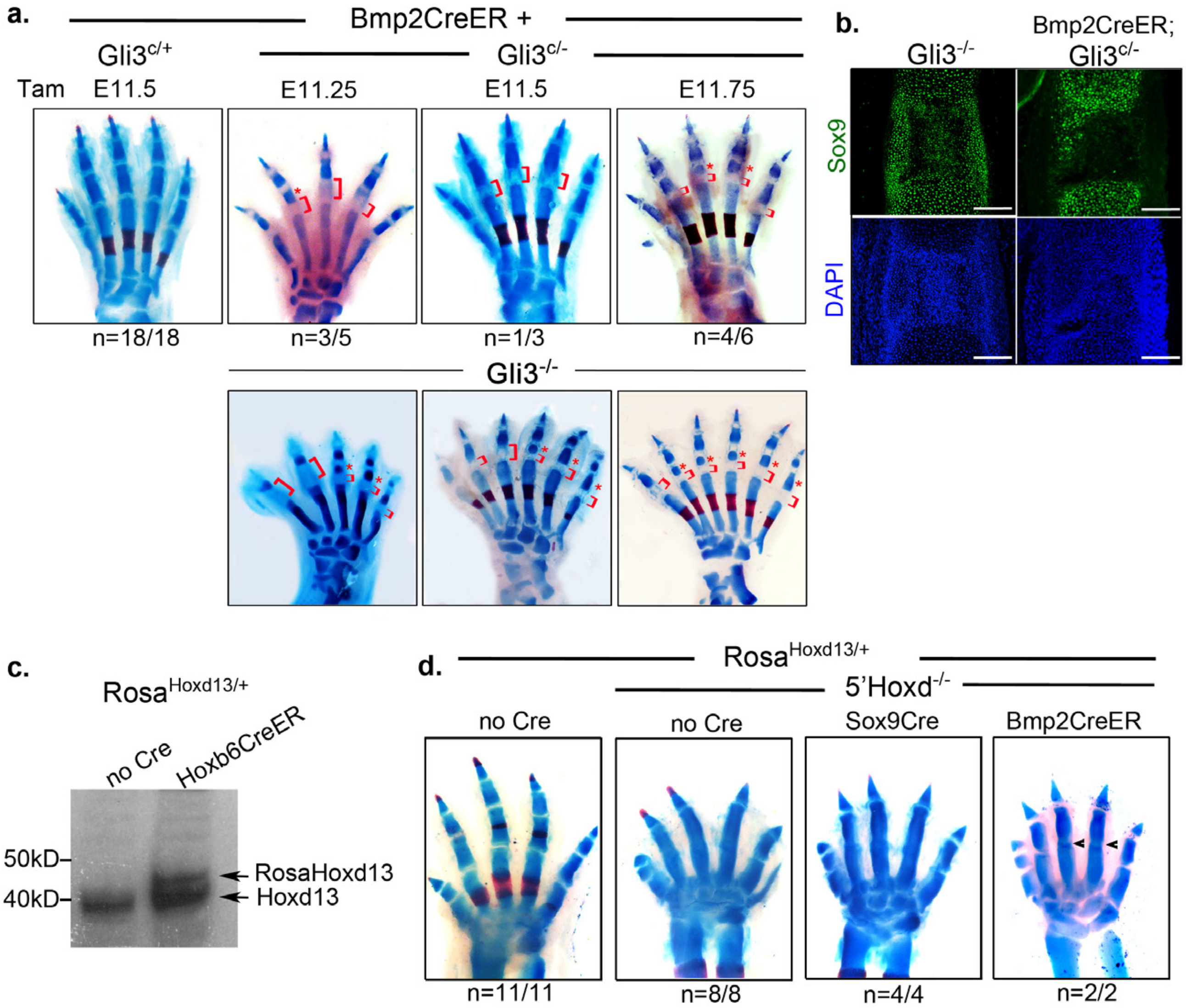

Supplementary Figure 4. Selective *Gli3* removal or *RosaHoxd13* over-expression in interdigits alters phalangeal joint formation.

a. Joint phenotype in *Gli3$^{c/-}$* digits after interdigit-specific excision by Bmp2CreER at different times (single tamoxifen dose, 3mg). Expanded P1 interzones (brackets) are evident, albeit less broadly, even after tamoxifen-activated excision at E11.75. * highlights variable formation of partial P1 elements. Stage-matched *Gli3$^{-/-}$* embryos show similar P1 element and joint phenotypes.

b. Sox9 immunofluorescence and Dapi nuclear staining in sections of E17.5 digit P1 regions from *Gli3$^{c/-}$* following interdigital *Gli3* removal by Bmp2CreER (3mg tamoxifen,

E11.5) compared to *Gli3*$^{-/-}$ reveals extended cellular interzone with reduced Sox9 in both mutants. Scale bars=100 mm.

c. Immunoblot comparing transgenic RosaHoxd13 expression to endogenous Hoxd13 in wild-type limb buds (E12.5) after activation by Hoxb6CreER in limb mesoderm (3mg tamoxifen, E10.5). An antibody selective for the Hoxd13 paralog was used (gift from S. Stadler). RosaHoxd13 contains an N-terminal Flagx3 epitope, shifting its molecular weight.

d. Partial rescue of digit joint formation (n=2/2; arrowheads) in *5'Hoxd*$^{-/-}$ with wild-type *Gli3* levels after selective interdigit activation of *Rosa*$^{Hoxd13/+}$ transgene by Bmp2CreER (3mg tamoxifen, E11.5). Activation of *Rosa*$^{Hoxd13/+}$ in interzone/chondro-progenitors by Sox9Cre has no effect on joint phenotype.

Number of embryos examined indicated below relevant figure panels (in **a, d**).

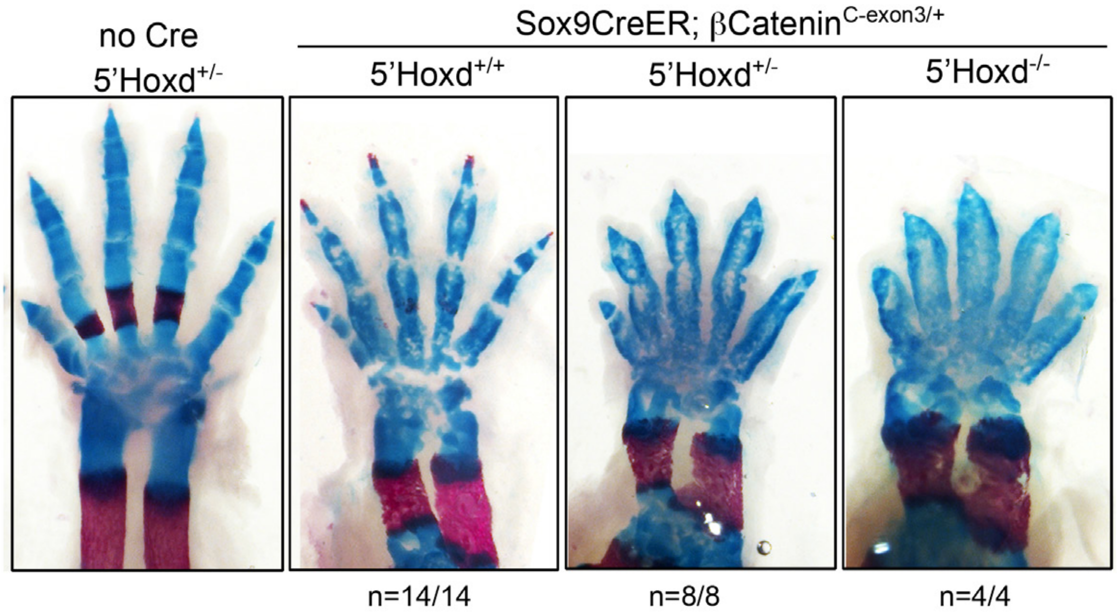

Supplementary Figure 5. Canonical Wnt pathway activation in interzone progenitors fails to restore *5'Hoxd$^{-/-}$* digit joints.

E17.5 skeletal stains show that activation of a stabilized βcatenin allele (exon3-deletion; *Catnb$^{C\text{-}exon3/+}$*) in interzone/chondro-progenitors by Sox9CreER (3mg tamoxifen at E11.25) fails to restore joint formation in *5'Hoxd$^{-/-}$* digits, although chondrogenesis is impaired. Note that joints are still apparent in control digits expressing the stabilized βcatenin. Number of embryos examined indicated below figure panels.

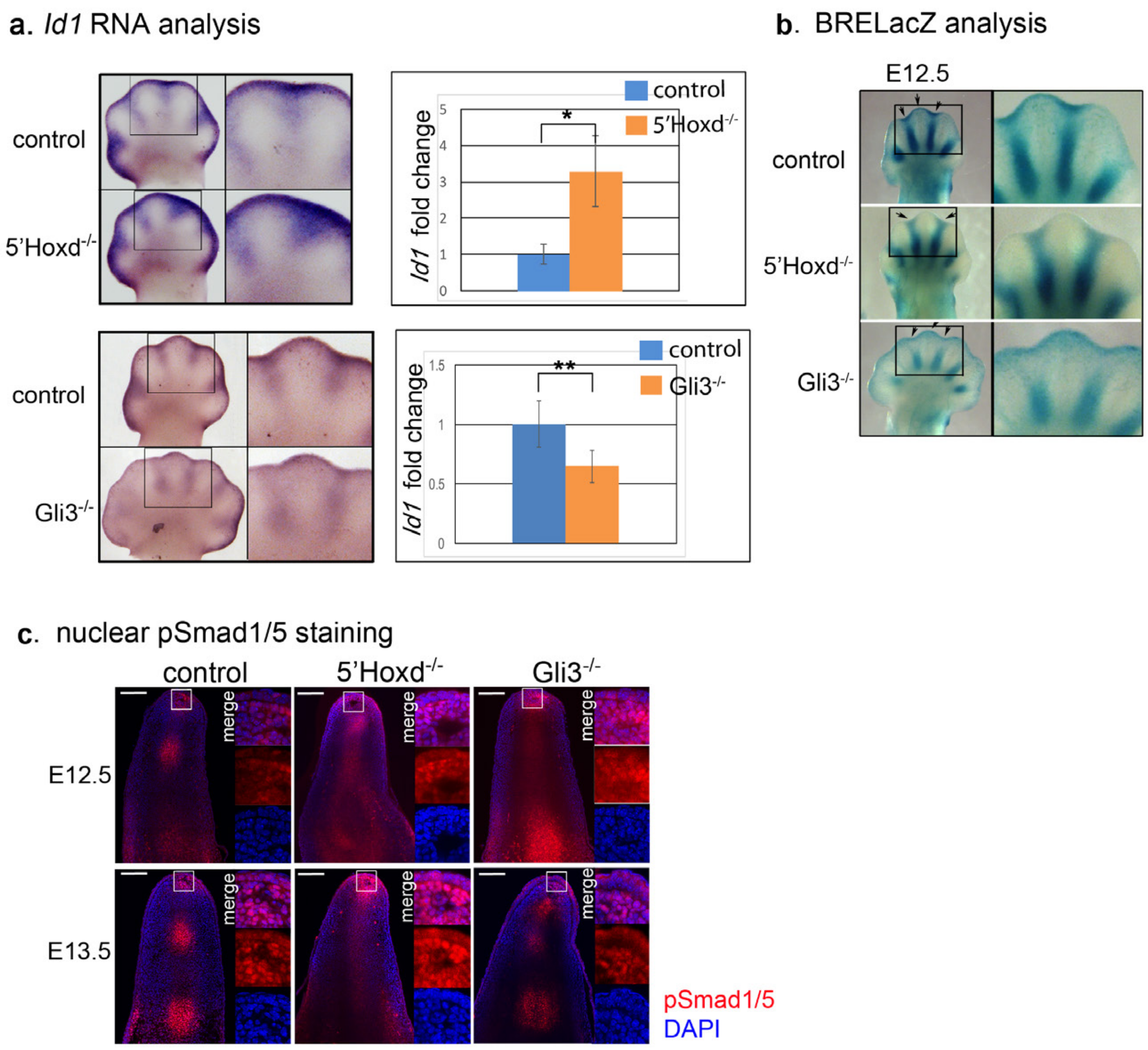

Supplementary Figure 6. Altered Bmp activity in *5'Hoxd*$^{-/-}$ and *Gli3*$^{-/-}$ distal digit regions.

a. Whole mount RNA analysis of the direct Bmp target *Id1* shows elevated levels in *5'Hoxd*$^{-/-}$ and reduced levels in *Gli3*$^{-/-}$ E12.5 distal digit tips (boxed areas enlarged to right of panels). qPCR analysis of posterior distal digital plate in *5'Hoxd*$^{-/-}$ (n=3) or anterior distal digital plate in *Gli3*$^{-/-}$ (n=4) compared to the equivalent control limb bud regions (dissections done as in diagrams shown in Supplementary Figure 3b above) confirms *in situ* expression changes (*p=0.03, **p=0.005; 2-tailed Student's t test). Error bars represent s.d.

b. Bmp activity around distal digit tips measured by BRELacZ reporter activation in control (wild type), *5'Hoxd*$^{-/-}$ and *Gli3*$^{-/-}$ digits at E12.5. Arrows highlight changes around the distal

tip regions compared to controls; boxed regions are enlarged in insets to right.

c. pSmad1/5 immunofluorescence in control and mutant E12.5 and E13.5 digits (from Figure 3d) with high magnification images of boxed areas at distal digit tip (panels to right) showing DAPI nuclear staining overlay (merge) and nuclear localization of pSmad1/5 immunofluorescence. Scale bars=100um.

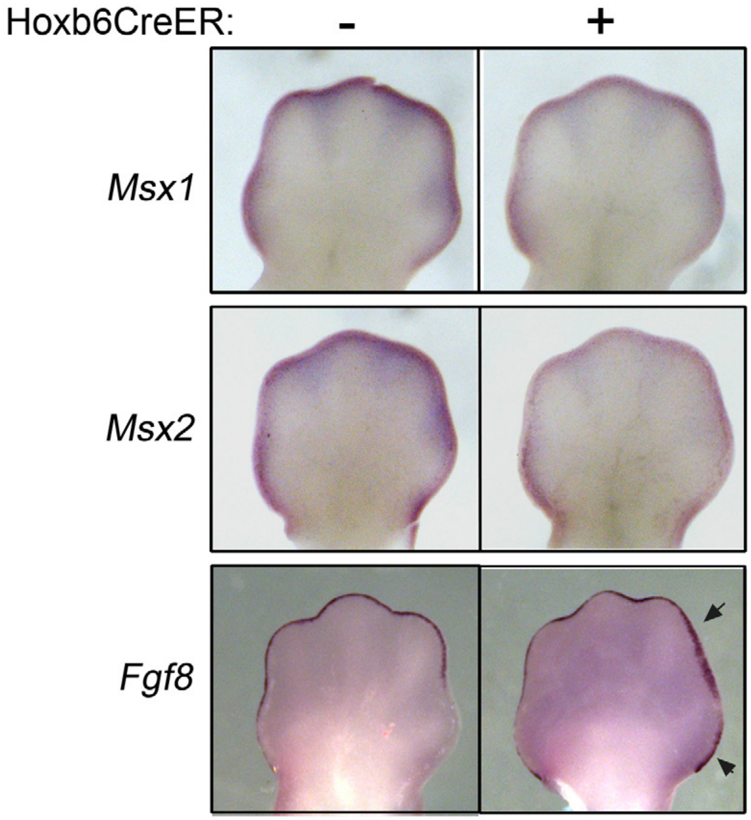

Supplementary Figure 7. *RosaGrem1* activation reduces Bmp activity and increases AER-*Fgf8*.

Reduced expression of direct Bmp target *Msx1* and *Msx2* RNAs in E12.5 forelimb buds and enhanced AER-*Fgf8* RNA expression (arrows) following mesenchymal *RosaGrem1* activation at E10.75 (Hoxb6CreER+; 3mg tamoxifen-single dose), confirming efficacy of Bmp-antagonist activation. Anterior side at left in all panels.

mHoxd11_F	CCGCAGCCTCTAACTTCTACA
mHoxd11_R	GCCTCGTAGAACTGATCAAAGC
mHoxd12_F	CTCTTGCCTGCGATCTTCACT
mHoxd12_R	GAATTCATTGACCAGGAATTCGTT
mHoxd13_F	AGGTGTACTGTGCCAAGGATCAG
mHoxd13_R	AAGCCACATCTCCTGGAAAGG3
mGli3_F	ATTCCCGTAGCAGCTCTTCA
mGli3_R	TTGCTGTCGGCTTAGGATCT
mId1_F	CTGAACGGCGAGATCAGTG
mId1_R	TTTTCCTCTTGCCTCCTGAA
mVimentin_F	GAACCTGAGAGAAACTAACC
mVimentin_R	ATGCTGAGAAGTCTCATTG
cHoxd11_F	AACAGTTCAGCAGTTCCTC
cHoxd11_R	TCATCCTTCGATTCTGGAAC
cHoxd12_F	AGTCCTTTGTTGGAAATGTG
cHoxd12_R	ATTGGGAAAATAAAAGGAATCTG
cHoxd13_F	AAAGTACATGGACGTCTCC
cHoxd13_R	TAGGAGGACACTTCTTTAGC
cGli3_F	CGCTACCATTATGAACCATC
cGli3_R	GAAGAATCTGAATATGTTGGGC
cVimentin_F	TGCCCTTAAAGGAAGTAATG
cVimentin_R	ATAGTGTCCTGGTAGTTAGC

Supplementary Table 1. Primer sequence pairs used for qPCR**.**

m, mouse; c, chick; F, forward primer 5’-3’; R, reverse primer 5’-3’

Supplementary References

1. Buscher, D., Bosse, B., Heymer, J., & Ruther, U. Evidence for genetic control of Sonic hedgehog by Gli3 in mouse limb development. *Mechanisms of Development* 62, 175-182, doi;10.1016/S0925-4773(97)00656-4 (1997).

2. Nelson, C.E., et al. Analysis of Hox gene expression in the chick limb bud. *Development* 122, 1449-1466 (1996).
